# A Neural Model of Intrinsic and Extrinsic Hippocampal Theta Rhythms: Anatomy, Neurophysiology, and Function

**DOI:** 10.3389/fnsys.2021.665052

**Published:** 2021-04-28

**Authors:** Stephen Grossberg

**Affiliations:** Center for Adaptive Systems, Department of Mathematics and Statistics, Department of Psychological and Brain Sciences, and Department of Biomedical Engineering, Boston University, Boston, MA, United States

**Keywords:** hippocampus, entorhinal cortex, learning, grid cell, place cell, spatial navigation, adaptively timed learning, Adaptive Resonance Theory

## Abstract

This article describes a neural model of the anatomy, neurophysiology, and functions of intrinsic and extrinsic theta rhythms in the brains of multiple species. Topics include how theta rhythms were discovered; how theta rhythms organize brain information processing into temporal series of spatial patterns; how distinct theta rhythms occur within area CA1 of the hippocampus and between the septum and area CA3 of the hippocampus; what functions theta rhythms carry out in different brain regions, notably CA1-supported functions like learning, recognition, and memory that involve visual, cognitive, and emotional processes; how spatial navigation, adaptively timed learning, and category learning interact with hippocampal theta rhythms; how parallel cortical streams through the lateral entorhinal cortex (LEC) and the medial entorhinal cortex (MEC) represent the end-points of the What cortical stream for perception and cognition and the Where cortical stream for spatial representation and action; how the neuromodulator acetylcholine interacts with the septo-hippocampal theta rhythm and modulates category learning; what functions are carried out by other brain rhythms, such as gamma and beta oscillations; and how gamma and beta oscillations interact with theta rhythms. Multiple experimental facts about theta rhythms are unified and functionally explained by this theoretical synthesis.

## 1. Introduction: Hippocampal Learning, Theta Rhythm, and Cholinergic Modulation

The topic of hippocampal theta rhythms combines several basic themes about how our brains work. These themes include, in varying degrees of generality:

• How theta rhythms were discovered;
• How theta rhythms organize brain information processing into temporal series of spatial patterns;
• How distinct theta rhythms occur within area CA1 of the hippocampus and between the septum and area CA3 of the hippocampus;
• What functions theta rhythms carry out in different brain regions, notably CA1-supported functions like learning, recognition, and memory that involve visual, cognitive, and emotional processes;
• How spatial navigation, adaptively timed learning, and category learning interact with hippocampal theta rhythms;
• How parallel cortical streams through the lateral entorhinal cortex (LEC) and the medial entorhinal cortex (MEC) represent the end-points of the What cortical stream for perception and cognition, and the Where cortical stream for spatial representation and action;
• How the neuromodulator acetylcholine interacts with the septo-hippocampal theta rhythm and modulates category learning;
• What functions are carried out by other brain rhythms, such as gamma and beta oscillations; and
• How gamma and beta oscillations interact with theta rhythms.

Before discussing theta rhythms *per se*, I will set the stage by discussing some of the main anatomical, neurophysiological, and functional properties of the hippocampus with which it interacts.

### 1.1. Hippocampal Place Cells

**FIGURE 1:**
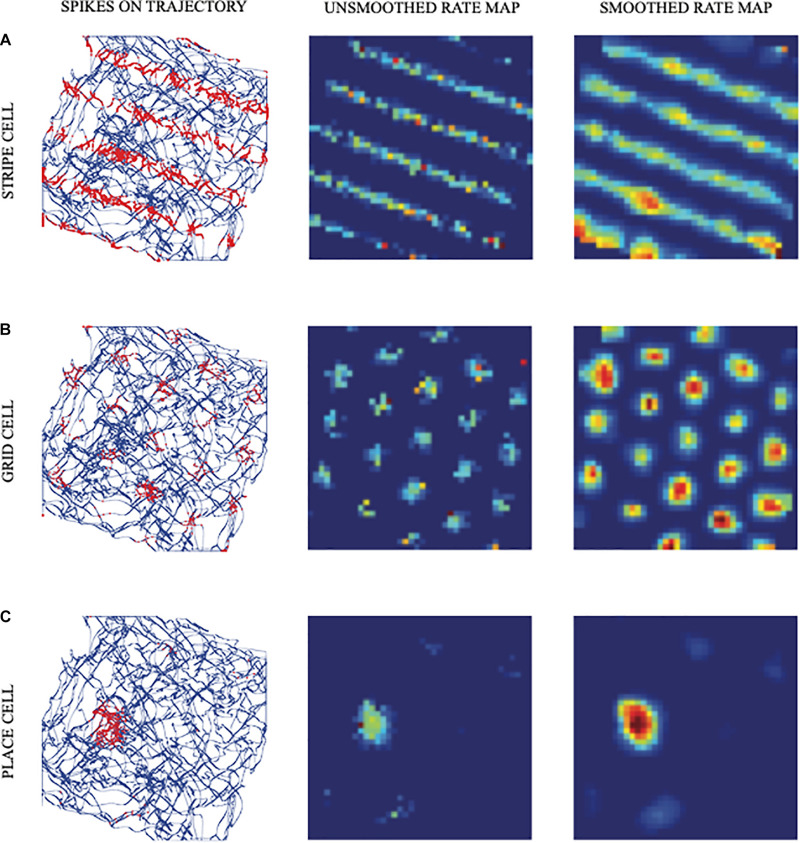
Simulated responses of illustrative model **(A)** stripe cells, **(B)** grid cells, and **(C)** place cells in the spiking self-organizing map (SOM) model. The first column shows the realistic trajectory of the animat (blue lines and curves) on which are superimposed spikes (red dots) of firing cells. The second and third columns show unsmoothed and smoothed spatial rate maps of the cells, respectively. Color coding from blue (minimum) to red (maximum) firing generates each rate map (reprinted with permission from Pilly and Grossberg, 2013).

Brain circuits within the hippocampus (HC) interact with the MEC to regulate spatial learning and memory (Morris et al., 1982; Davis et al., 1992; Parron and Save, 2004). Particular cell types within these brain regions arise through this learning process to support spatial navigation. Perhaps the most important of these cell types are the place cells which fire whenever rats, or multiple other animals, are at particular localized regions, or “places,” within an environment (Figure 1C; O’Keefe and Dostrovsky, 1971). Place cells can also exhibit multiple firing fields when animals navigate in large spaces (Fenton et al., 2008; Henriksen et al., 2010; Park et al., 2011). Different place cells fire at different locations in an environment, so that the ensemble of all place cells enables an animal to localize itself in an environment, and to navigate through it.

The classical article of O’Keefe and Dostrovsky (1971) reported that place cells receive two kinds of inputs: one conveying information about the sensory context experienced from a given place, and the other from a navigational, or path integration, system that tracks relative positions in the world by integrating self-movement angular and linear velocity estimates for instantaneous rotation and translation, respectively. An important open problem is to explain how sensory context and path integration information are combined in the control of navigation (Etienne et al., 1996; Gothard et al., 1996; Chen et al., 2013). Path integration information is generated by an animal’s movements, including its distance and direction relative to a start location (Loomis et al., 1999). Path integration is the basis of “dead reckoning” which was used by sailors for hundreds of years to estimate the location of their ship using information about its speed, travel time, and direction when visible landmarks were not available.

### 1.2. Entorhinal Grid Cells

Entorhinal grid cells provide inputs to place cells in hippocampal area CA3. They occur in the superficial layers of MEC and exhibit one of the most remarkable receptive fields of any cell type in the brain. Indeed, each grid cell can fire in multiple locations that may form a regular hexagonal grid (Figure 1B; Hafting et al., 2005). It is known that path integration inputs are a primary cause of these grid cell properties (McNaughton et al., 2006).

Cells in a population of grid cells at a given dorsoventral location in rat MEC exhibit receptive fields that are offset from each other (Hafting et al., 2005). As the dorsolateral MEC is traversed from its dorsal to its ventral end, grid cell receptive field sizes increase, as does the spacing between these fields, on average (Sargolini et al., 2006; Brun et al., 2008; Stensola et al., 2012).

### 1.3. Place Cells Can Represent Spaces Which Are the Least Common Multiple of Their Grid Cell Scales

**FIGURE 2:**
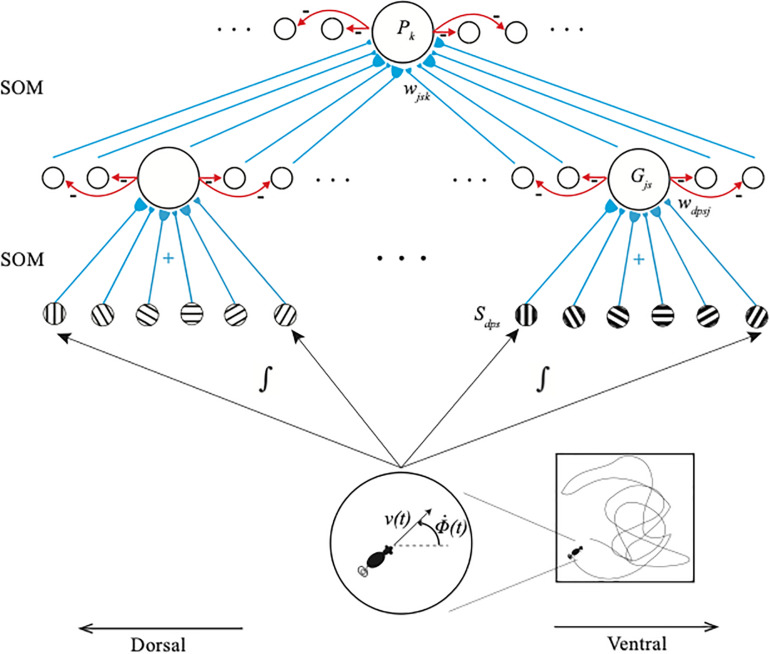
The GridPlaceMap is defined by a hierarchy of self-organizing maps (SOM) whereby grid cells and place cells are learned and activated. Stripe cells S_dps_ occur in the deeper layer of medial entorhinal cortex (MEC), grid cells G_js_ occur in layer II of MEC, and place cells P_k_ occur in hippocampal area CA3. Multiple stripe cells drive learning of individual grid cells, and multiple grid cells drive learning of individual place cells. Place cells can thereupon represent movements in large spaces in response to internally generated linear velocity [v(t)] and angular velocity [Φ(t)] movements through the environment. Bigger stripe fields and spacings occur from dorsal to ventral positions in the model (reprinted with permission from Grossberg and Pilly, 2014).

These grid cell properties have led to the suggestion that the receptive field of a place cell that fires at a given position may be derived by combining grid cells with multiple spatial phases and scales that are coactive at that position, thereby allowing a place cell to represent a spatial scale that is much larger than the spatial scales of the individual grid cells that input to it (O’Keefe and Burgess, 2005; McNaughton et al., 2006; Gorchetchnikov and Grossberg, 2007). Indeed, the GridPlaceMap model (Figure 2) and its earlier variants explains why the spatial scale of a place cell can be as large as the *least common multiple* of the grid cell scales that drive it (Gorchetchnikov and Grossberg, 2007; Grossberg and Pilly, 2012, 2014; Mhatre et al., 2012; Pilly and Grossberg, 2012).

For example, grid cell spatial scales of 40, 50, and 60 centimeters that input to place cells can endow the place cells with a spatial scale of 6 m. If the converging grid cell spatial scales are 50, 60, and 70 centimeters, then the place cell spatial scale is 21 m. Remarkably, grid cell spatial scales that are chosen to be 41, 53, and 59 centimeters can generate a place cell spatial scale of 1.282 km (Gorchetchnikov and Grossberg, 2007)! Whereas, the spatial scales of individual grid cells are too small to control navigation in real-world spaces, the spatial scales of place cells can do so.

### 1.4. Stripe Cells

**FIGURE 3:**
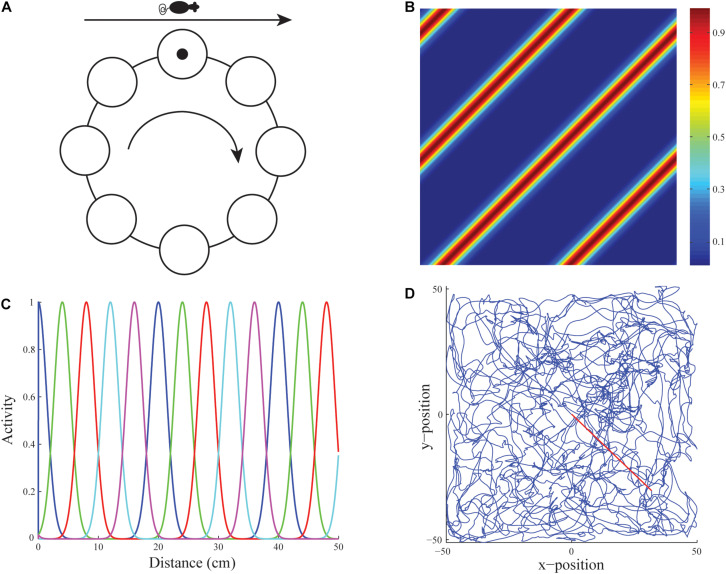
Linear velocity path integration. **(A)** Linear velocity path integration signals input to a ring attractor neural circuit that translates them into an activity bump that moves from cell to cell along the ring. **(B)** Firing rate map of an illustrative stripe cell with a spacing of 35 cm whose firing fields respond to translational movement with a component along either 135^o^ or −45^o^. **(C)** Activities of stripe cells of five different spatial phases (see colors) as a function of displacement from the origin along their preferred direction. **(D)** Real rat trajectory from Sargolini et al. (2006) of ∼10 min in a 100 cm × 100 cm environment that was used to train the model. The red segment depicts the straight path prefixed to the original trajectory to ensure the animat starts from the midpoint of the environment (reprinted with permission from Pilly and Grossberg, 2012).

Grid cells are learned within the GridPlaceMap model in response to inputs from multiple *stripe cells* (Sargolini et al., 2006; Krupic et al., 2012) each of which fires selectively in response to movements in a given direction with its own spatial scale and spatial phase. Each stripe cell codes the distance from a starting position by integrating the linear velocity of the navigator. In the GridPlaceMap model, stripe cells are organized into ring attractors (Figure 3A). All the stripe cells in a given ring attractor are tuned to movement along the same direction. Because they occur at different positions in the ring attractor, different stripe cells fire at different spatial phases (Figure 3C). An activity bump which integrates the distance traveled moves from one stripe cell to the next on the ring attractor as the animal moves in its prescribed direction (Figure 3A).

The name *stripe cell* describes the periodic directionally selective activations of these cells as the environment is navigated. Because the activity bump moves around the ring attractor as the model animal, or animat, moves in a given direction, a sufficiently long excursion executes a complete cycle, which activates the same stripe cell again (Figure 3B). This distance determines the spatial scale of that ring attractor. Parallel activations of multiple stripe cell ring attractors, each responding selectively to a different spatial scale and directional preference, implicitly represent the animal’s position in the environment. Multiple stripe cells input to a given grid cell, just as multiple grid cells input to a given place cell (Figure 2). The GridPlaceMap model explains how learning prunes which stripe cells can activate particular grid cells to form their hexagonal spatial receptive fields, as it also prunes which grid cells can activate particular place cells.

### 1.5. A Parsimoniously Designed Hierarchy of Self-Organizing Maps Defines Grid and Place Cells

The GridPlaceMap model has a remarkably parsimonious and elegant design. Both grid cell and place cell receptive fields, despite their radically different properties, develop by detecting, learning, and remembering the most frequent and energetic co-occurrences of their inputs. The same self-organizing map (SOM) circuits automatically compute these co-occurrences to learn grid cell and place cell receptive fields, albeit at different levels of the learning hierarchy (Figure 2). SOMs occur in multiple parts of the brain, where they learn perceptual, cognitive, motor, or spatial recognition categories (Grossberg, 2021). In the GridPlaceMap model, place cells are spatial categories. More about how SOM models work will be described in the discussion in Section 3.3 of how SOMs learn perceptual and cognitive recognition categories.

All the inputs that drive grid cell learning arise from ring attractor circuits, as noted in Section 1.4. One type of ring attractor circuit processes linear velocity inputs that input to stripe cells. The other ring attractor circuit processes angular velocity inputs that input to head direction cells, which modulate the directional selectivity of different stripe cells.

### 1.6. Temporal-to-Spatial: Learning a Dorsoventral Gradient of Increasing Grid Cells and Time Cells

Populations of stripe cells with a given spatial period that exhibit multiple directional preferences and spatial phases initially project with random adaptive weights to cells in the category learning layer of a SOM (Figure 2). Learning tunes these weights until the SOM cells become grid cells of fixed spatial scales, just as such learned tuning responds to the emerging grid cells by creating SOM cells at the next processing stage with place cell receptive fields.

*In vivo*, grid cells and place cells of increasing spatial scale are found along a dorsoventral gradient. The GridPlaceMap model explains how stripe cells of different spatial scales learn to activate different locations along the dorsoventral axis in layer II of MEC, thereby triggering learning of grid cells with increasing spatial scales. The selection of different grid cell spatial scales on the dorsoventral gradient is driven by a gradient in the *rates* at which cells respond along the dorsoventral gradient, from fast to slow. Cells that respond more quickly are active for shorter time intervals than cells that respond more slowly.

A longer duration of sampling enables each grid cell to sample stripe cells over a larger spatial scale. The duration of sampling thus covaries with the spatial scale of the learned grid cells. Likewise, multiple entorhinal grid cell scales combine through learning to give rise to a dorsoventral gradient of hippocampal place cells, each of which can represent larger spaces than can an individual grid cell. Due to these temporal-to-spatial conversions, the SOM model shows how the dorsoventral gradient in response rates develops a spectrum of grid cell scales and place cell scales. It is thus called the Spectral Spacing model.

Remarkably, the response rate gradient for spatial learning is computationally homologous to a rate gradient that had, 20 years earlier, been used to explain hippocampal data about adaptively timed learning (Grossberg and Schmajuk, 1989; Grossberg and Merrill, 1992, 1996). The model for adaptively timed learning was called a Spectral Timing model because its different cell populations respond with a “spectrum” of different rates whose activities peak at different times, and whose total *population* activity can learn to peak hundreds of milliseconds after their onset. That is why the GridPlaceMap model of grid cell learning is called a Spectral Spacing model. The rate gradient for spatial learning occurs in the MEC and its hippocampal projections. The rate gradient for temporal learning occurs in the LEC and its hippocampal projections.

One of the most striking properties of the Spectral Timing model is its Weber Law property whereby cells in the spectrum that respond at larger times also respond with longer durations. This prediction was strongly confirmed by the discovery, 20 years after it was made, of *time cells* in the hippocampus by MacDonald et al. (2011). Indeed, MacDonald et al. (2011) write: “…the mean peak firing rate for each time cell occurred at sequential moments, and the overlap among firing periods from even these small ensembles of time cells bridges the entire delay. Notably, the spread of the firing period for each neuron increased with the peak firing time…” (p. 3). Their “small ensembles of time cells” are the cells in the spectrum, and the “spread of the firing period…increased with the peak firing time” is the Weber Law property. For a recent synthesis of hippocampal timing processes, see Banquet et al. (2020).

In summary, parallel dorsoventral gradients in the rates that cells respond within the entorhinal–hippocampal system may create multiple smaller spatial and temporal scales in the entorhinal cortex that can be fused into larger spatial and temporal scales in the hippocampal cortex, notably scales that are large enough to control adaptive behaviors during spatial navigation in large enclosures. The mechanistic homology between these spatial and temporal mechanisms suggests why they may occur side-by-side in MEC and LEC dorsoventral gradients through entorhinal cortex into the hippocampus.

Spatial representations in the dorsal, or Where, cortical stream for spatial representation and action go through postrhinal cortex and MEC on their way to hippocampal cortex, whereas object representations in the ventral, or What, cortical stream for perception and object recognition go through perirhinal cortex and LEC on their way to hippocampal cortex (Hargreaves et al., 2005; Norman and Eacott, 2005; Aminoff et al., 2007; Kerr et al., 2007; Eichenbaum and Lipton, 2008; van Strien et al., 2009; LaChance et al., 2019; Nilssen et al., 2019; Sethumadhavan et al., 2020), where they are merged.

### 1.7. Episodic Learning and Memory

These parallel and homologous spatial and temporal representations may clarify the role of hippocampus in supporting episodic learning and memory (Tulving, 1972; Tulving and Thomson, 1973), since each episode in memory consists of specific spatio-temporal combinations of cues, imagery, and behaviors.

Episodic learning combines information about particular *sequences* of object and spatial information. The importance of sequential processing during both episodic learning and spatial navigation have led Buzsáki (2005, p. 831) to suggest that “Learning of sequentially presented or inspected random items in an episodic task is formally identical to the coding of sequential places in a linear (1-dimensional) route” and, moreover, that “formation of episodes and neuronal representations of 1-dimensional routes require a temporal metric that we identify with the theta period.” Buzsáki and Moser (2013, p. 130) have similarly suggested the hypothesis that “mechanisms of memory and planning have evolved from mechanisms of navigation in the physical world and hypothesize that the neuronal algorithms underlying navigation in real and mental space are fundamentally the same. We review experimental data in support of this hypothesis and discuss how specific firing patterns and oscillatory dynamics in the entorhinal cortex and hippocampus can support both navigation and memory.”

The brain processes that underly the proposed homology between navigation and memory have been modeled in a series of studies over the years. One extensive set of psychophysical experiments about the storage and learning of sequential information has studied how context-sensitive searches are carried out for desired objects in scenes, under the rubric of *contextual cueing*. For example, when we are in our kitchen and see a refrigerator and a stove at particular positions, we then expect to see our sink at a prescribed different position. The psychophysical literature about contextual cueing describes how sequentially experienced objects and positions contribute to such expectations, and guide efficient searches to discover and act upon desired goal objects (e.g., Chun and Jiang, 1998; Olson and Chun, 2002; Brockmole et al., 2006; Jiang et al., 2006). The contextual cueing literature hereby provides a well-organized database for studying the learning of episodic memories, using brain regions that are also implicated in the learning of navigational trajectories.

The ARTSCENE Search model (Huang and Grossberg, 2010) simulates how sequences of experienced spatial locations and objects are temporarily stored in spatial and object working memories, before they trigger learning of spatial and object sequence categories, or list chunks, which in turn learn spatial and object priming signals whereby to predict and control what events to expect and act upon in that sequential context. These processes are modeled using interactions between the perirhinal and parahippocampal cortices, prefrontal cortex, temporal cortex, and parietal cortex to simulate key psychophysical data from contextual cueing experiments that are reviewed and simulated in Huang and Grossberg (2010). The ARTSCENE Search, Spectral Spacing, and START models (see Section 5.1) may in the future be fused to provide a foundation on which to build a more complete theory of episodic learning and memory.

### 1.8. Shared mGluR Dynamics of Spectral Timing in Hippocampus, Cerebellum, and Basal Ganglia?

To the present, spectral timing processes, with similar biophysics and circuitry, have been modeled in the hippocampus, cerebellum, and basal ganglia (Fiala et al., 1996; Brown et al., 1999, 2004). The proposed mechanism of spectral timing in the cerebellum has been supported by biophysical data concerning the calcium dynamics of the metabotropic glutamate receptor (mGluR) system that enables spectral timing to bridge temporal delays of hundreds of milliseconds (Fiala et al., 1996). The most parsimonious prediction is that a similar mechanism holds in all cases of spectral timing throughout the brain.

### 1.9. Linking Theta Rhythm to Grid Cells and Place Cells

The above brief review of grid cell and place cell properties has been given because brain rhythms, including the theta rhythm, are not disembodied abstract oscillators. Rather they modulate particular brain processes. It is known, for example, that a theta rhythm plays a key role in modulating the normal processing of entorhinal grid cells and hippocampal place cells. In this regard, Stensola et al. (2012, p. 72) have shown that grid cells along the dorsoventral axis “cluster into a small number of layer-spanning anatomically overlapping modules with distinct scale, orientation, asymmetry and theta-frequency modulation.” These grid cell modules are distributed across wide regions along the dorsoventral axis with substantial overlaps among the different clusters.

There is also an intrinsic theta rhythm generated endogenously within the hippocampus (Vanderwolf, 1988; Smythe et al., 1992; Bland and Colom, 1993), in addition to an extrinsic theta rhythm that is transmitted to hippocampal projections from the medial septal (MS) area in the basal forebrain, and that generates and maintains a theta rhythm in the hippocampal and parahippocampal areas (Vertes and Kocsis, 1997) via reciprocal interactions among GABAergic interneurons (Tóth et al., 1993; Wang, 2002). This theta rhythm can be reduced by inactivating the MS, which also disorganizes the hexagonal spatial firing patterns of grid cells (Brandon et al., 2011; Koenig et al., 2011). During MS inactivation, affected grid cells tend to code the rat’s head direction, in keeping with how inputs from head direction cells form stripe cell, and thus grid cell, receptive fields (Figure 1). These and other properties of the intrinsic and extrinsic theta rhythm will be explained below.

## 2. Discovery of Theta Rhythm and Basic Properties

### 2.1. Behaviors During Which Theta Rhythm Occurs

Winson (1974) provides an excellent account of when theta rhythms were first reported. Walter and Walter (1949) reported a theta rhythm in the frequency range of 3–7 cycles per second in their EEG scalp recordings of human subjects. Green and Arduini (1954) reported an “arousal reaction” in this frequency range within the hippocampus of the rabbit in response to natural sensory stimulation or electrical stimulation. Behavioral correlates of the theta rhythm were later recorded in several species (Bennett, 1971; Winson, 1972) including the rat, where a theta rhythm is found only if the animal carries out voluntary movements like exploration, as well as during paradoxical sleep (Vanderwolf, 1969).

Whishaw and Vanderwolf (1973) extended the list of behaviors that occur during a theta rhythm, and the species in which they occur. As they noted on p. 461, “slow electrical activity was recorded from the dorsal hippocampus in rats during running in a motor-driven wheel, swimming, conditioned avoidance (running, jumping), lever pressing for food and sleep, and in cats during walking in a treadmill, eating and lapping milk. Large-amplitude clear rhythmical slow activity (RSA) was recorded from the hippocampus proper and smaller amplitude RSA and fast activity was recorded from the dentate gyrus-CA 4 area…Increases in RSA frequency and amplitude during paradoxical sleep were associated with muscular twitches, suggesting that forebrain motor mechanisms were activated. The results are interpreted in support of the idea that RSA is related to higher level control of voluntary movement.”

The authors noted additionally on p. 477 that “previous reports on the relation between hippocampal RSA and behavior were also confirmed. In the experiments with rats it was found that RSA was present in the pyramidal cell layer in all situations at all times when the animals walked, ran, jumped, etc. (voluntary movement), but that RSA was absent if the animals chewed food, washed their faces, etc. (automatic movement). The results also indicated that the mere repetition of the same motor act does not lead to a disappearance of hippocampal RSA, when such activity was initially present.”

The article by Winson (1978) reported, for example, on p. 160 that “rats learned, using distal room cues, to run to a goal on an elevated circular track starting from any position on the track. The goal was one of eight equidistant, recessed cups set around the track, the goal cup being distinguished from the others solely by its position in the room. After learning, electrolytic lesions were made in the medial septal nucleus eliminating hippocampal theta rhythm…Rats without theta rhythm were no longer able to perform the spatial task…” These results showed how contextual visual cues could interact with a learned hippocampal spatial map to efficiently guide reinforced navigational behavior. They also showed that an important source of the extrinsic theta rhythm that supports such learned spatial behavior is the medial septum.

### 2.2. Intrinsic Theta Rhythm and Cholinergically Modulated Extrinsic Theta Rhythm

Several articles also reported that the septal projection that drives the extrinsic theta rhythm is modulated by acetylcholine (e.g., Stewart and Fox, 1990; Huerta and Lisman, 1993). Huerta and Lisman (1993) also noted on p. 723 that “theta rhythm occurs during periods of learning…during these oscillations, synapses are in a state of heightened plasticity [that] is sensitive to the timing of incoming stimuli with respect to the oscillatory activity…” In contrast to septally driven theta rhythms, intrinsic theta rhythms may be generated entirely within the CA1 region of the hippocampus where their atropine-resistance shows that their theta generators are not dependent upon acetylcholine (Kramis et al., 1975; Goutagny et al., 2009).

The subsequent discussion will situate the above facts within a larger modeling context that mechanistically and functionally explains them and many others.

## 3. Septo-Hippocampal Theta Rhythm, Vigilance Control, and Grid and Place Cell Category Learning

The following facts and theoretical results clarify how cholinergic signals from the medial septum to the hippocampus influence the extrinsic theta rhythm. One measure of this effect is how disruption of the theta rhythm, or cholinergic modulation, or both influence the learned hexagonal spatial firing patterns of entorhinal grid cells.

**FIGURE 4:**
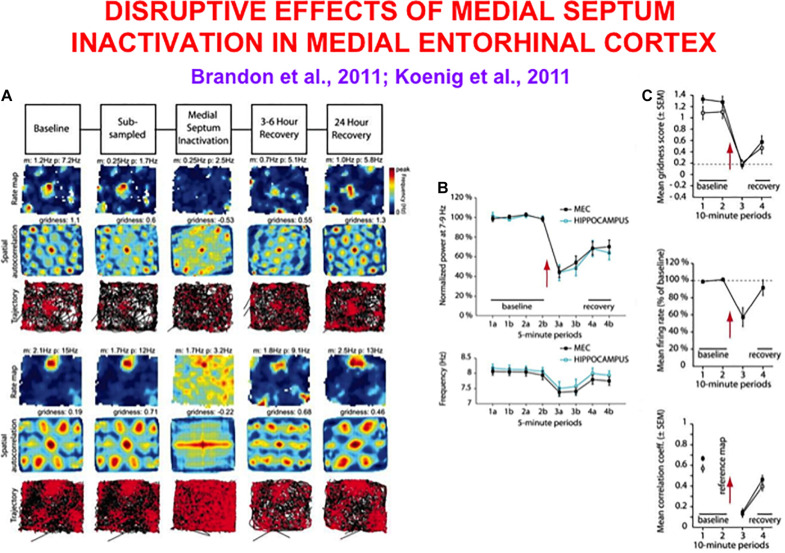
Data showing effects of medial septum (MS) inactivation on grid cells and network theta oscillations in medial entorhinal cortex (MEC). **(A)** Examples of disruption of the hexagonal grid structure of two grid cells (Brandon et al., 2011). **(B)** Temporal reduction in the power and frequency of network theta oscillations (Koenig et al., 2011). **(C)** Temporary reduction in the gridness score, mean firing rate, and spatial stability of grid cells (Koenig et al., 2011) (data reprinted with permission from Brandon et al., 2011 and Koenig et al., 2011).

Experiments that reduce the theta rhythm by inactivating the septum cause a correlated reduction in the hexagonal spatial firing patterns of grid cells (Brandon et al., 2011; Koenig et al., 2011). Figure 4 shows how septal inactivation causes the collapse of grid cell receptive fields, followed by their recovery after septal inactivation wears off. While the septum is inactivated, grid cells tend to code the rat’s head direction (Figure 4A, sixth row, middle curve).

Does inactivation of the septum lead to grid cell disorganization because the theta rhythm collapses, or because cholinergic modulation of grid cells collapses, or both? Some authors, particularly those who have espoused that oscillatory interference causes grid cell receptive fields, have concluded from these data that “spatial coding by grid cells requires theta oscillations” and “support a role of neuronal oscillations in the coding of spatial information” (Brandon et al., 2011). In other words, these authors proposed that their data support a mechanism of oscillatory interference in the creation of hexagonal grid fields.

### 3.1. Acetylcholine Modulates Vigilance Control of Cognitive, Motor, and Spatial Category Learning

Pilly and Grossberg (2013) proposed an alternative mechanistic explanation and presented data and other theoretical concepts that undermine an explanation based upon oscillatory interference. Their explanation emphasizes that signals from the septum to entorhinal grid cells typically release acetylcholine, and that acetylcholine modulates the learning of recognition categories via a process of *vigilance control* (Carpenter and Grossberg, 1987a; Grossberg and Versace, 2008). The data about collapse of grid cells due to septal inactivation are explained as a collapse of vigilance control that is needed to learn hippocampal *spatial* categories, with or without a theta rhythm. Vigilance control also modulates the learning of cognitive and motor categories, as discussed below. ART is also contrasted with continuous attractor and SOM grid cell models in Grossberg and Pilly (2014).

### 3.2. How Reduction of Cholinergic Input to Grid Cells Causes Their Collapse

In the case of the entorhinal-hippocampal system, the inactivation of the medial septum eliminates, or greatly reduces, the cholinergic input to grid cells (Mitchell et al., 1982). This reduction increases the conductances of leak potassium and slow and medium after-hyperpolarization channels (Madison et al., 1987; Müller et al., 1988; Klink and Alonso, 1997; Lape and Nistri, 2000). The rate of membrane depolarization is hereby slowed, leading to greater spike frequency adaptation and longer refractory periods. Grid cell firing and spatial periodicity are hereby greatly impaired because a controlled duration of grid cell sampling of stripe cell inputs is needed to learn, and maintain, grid cells with any given spatial scale.

**FIGURE 5:**
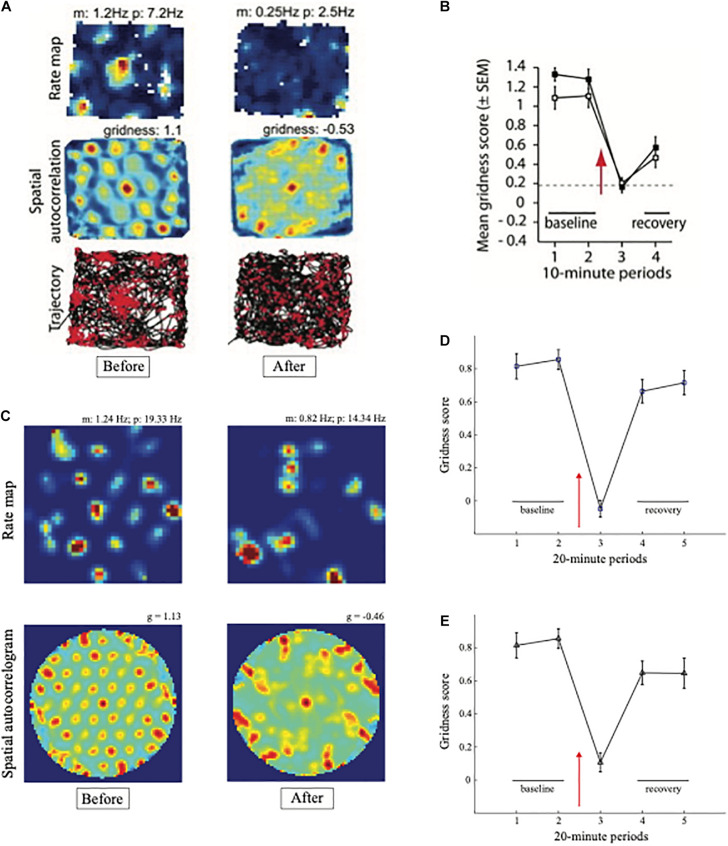
Data and computer simulation of effects of septal inactivation on grid cells. **(A)** Each row shows data and different data-derived measures of grid cell responsiveness, starting from the left with the baseline response to the middle column with maximal inhibition (Data reprinted with permission from Brandon et al., 2011). **(B)** Data showing the temporary reduction in the gridness scores during septal inactivation, followed by recovery (Data reprinted with permission from Koenig et al., 2011). **(C)** Simulation of gridness collapse due to reduction in cell response rates that mimic reduced cholinergic transmission. **(D)** Simulations of gridness score reduction by reducing cell response rates. **(E)** Simulations of gridness score reduction by changing leak conductance [simulations in **(C–E)** reprinted with permission from Grossberg and Pilly, 2014].

**FIGURE 6:**
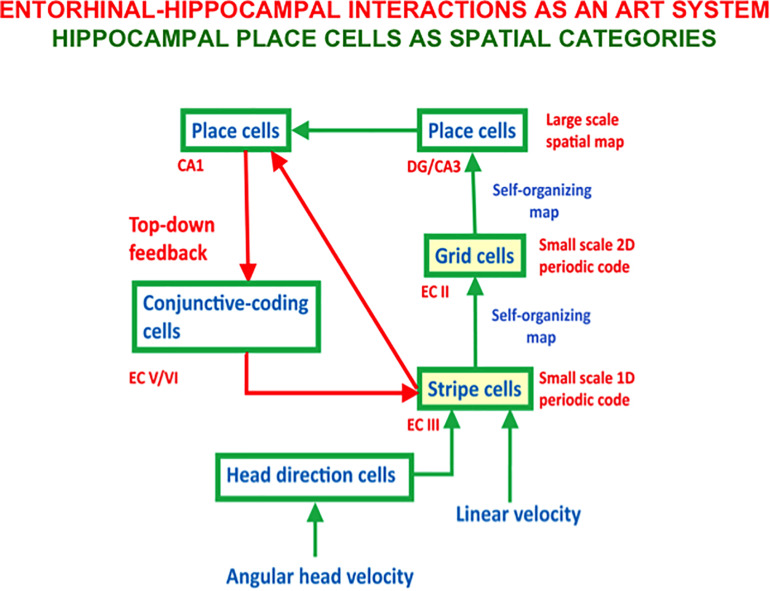
The entorhi nal-hippocampal system has properties of an Adaptive Resonance Theory, or ART, spatial category learning system. Hippocampal place cells learn spatial recognition categories in this system. Learned grid cell and place cell receptive fields are dynamically stabilized by top-down feedback that obeys the ART Matching Rule from CA1 place cells to entorhinal cortex (adapted with permission from Mhatre et al., 2012).

Model simulations in Figure 5 support these conclusions by showing spatial disorganization of grid fields as well as reductions in firing rate and spatial stability (Figure 5C) when septal inactivation is caused either by a temporary reduction in cell response rates (Figure 5D) or a temporary increase in leak conductances (Figure 5E). A head direction bias remains after the duration of path integration sampling is disorganized due to the remaining head direction inputs from stripe cells to grid cells in the GridPlace Map model of Figure 6.

Newman et al. (2014) summarized experimental evidence that are consistent with this explanation by demonstrating that “administration of the muscarinic antagonist scopolamine flattens the typically positive correlation between running speed and entorhinal theta frequency in rats…spatial tuning of grid cells was reduced following scopolamine administration. The tuning of head direction cells, in contrast, was not reduced by scopolamine” (p. 643). The authors go on to write “This is the first report to demonstrate a link between cholinergic function and grid cell tuning.” It also supports the prediction by Pilly and Grossberg (2013) of the existence of this link, and how it works.

Figure 6 also includes a top-down feedback pathway that dynamically stabilizes the learning of grid cells and place cells using an attentive matching process that obeys the ART Matching Rule, which will be described in the next section. The need for this feedback loop is clarified by the fact that place cells can develop in minutes, and some of them can retain their stability for months (Thompson and Best, 1990; Wilson and McNaughton, 1993; Muller, 1996; Ziv et al., 2013). However, a SOM does not have this property because learned SOM categories can undergo catastrophic forgetting. In the case of grid cells and place cells, this means, for example, that the grid cell receptive fields could drift through time. As noted below, Adaptive Resonance Theory, or ART, was introduced to explain how learned categories across the brain can dynamically stabilize their memories for many years.

### 3.3. Dynamically Stabilizing Learned Cognitive and Spatial Categories Using the ART Matching Rule

**FIGURE 7:**
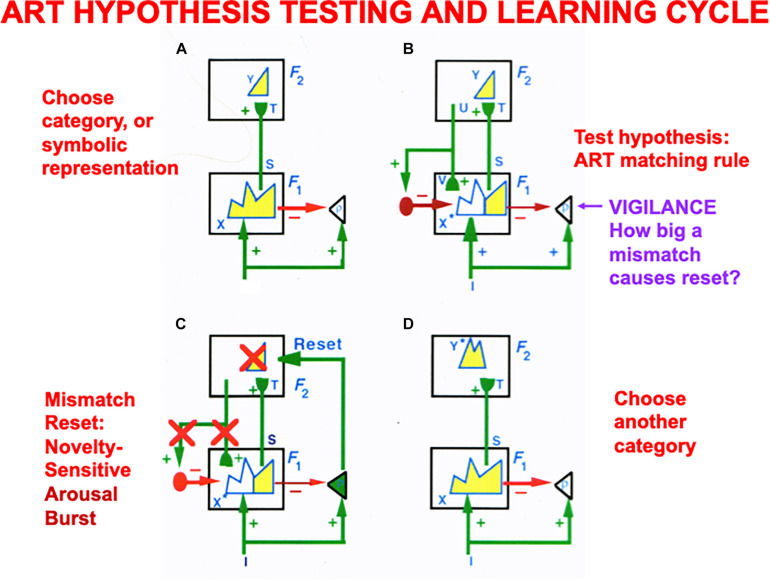
**(A)** The ART hypothesis testing and category learning cycle begins when an input pattern *I* is stored across feature detectors at level *F*_1_ as an activity pattern *X*, which is shown in yellow. For simplicity, arrays of connections between processing stages are represented by a single connection. While *X* is getting instated in *F*_1_, *I* also send excitatory signals via parallel pathways to the orienting system (the triangle with gain parameter *ρ* inside it). The gain parameter *ρ* is the *vigilance* parameter. As activity pattern *X* is instated in *F*_1_, it activates two output signal pathways: A bottom-up excitatory input pattern *S* to category level *F*_2_, and inhibitory inputs *ρI* to the orienting system. There are as many excitatory inputs as inhibitory inputs to the orienting system, because inputs *I* activate pattern *X*. The net input to the orienting system is *ρ/I/* – /*X/*, where // denotes the size of each total input. This net input is not positive because *ρ* ≤ 1, so the orienting system remains quiet. Before activating a category in *F*_2_, bottom-up signals *S* are multiplied by learned adaptive weights (in the hemispherical synapses) to generate the input pattern *T* to category level *F*_2_. Inputs *T* are contrast-enhanced and normalized within *F*_2_ by a recurrent shunting on-center off-surround network that activates and stores a small number of cells within *F*_2_ that receive the largest inputs. The chosen cells represent category *Y* that codes feature pattern at *F*_1_. **(B)** Category *Y* generates top-down signals *U* that are multiplied by adaptive weights to form a prototype, critical feature pattern, or expectation *V* of what learned feature pattern to attend at *F*_1_. Expectation *V* delivers an excitatory modulatory signal to *F*_1_ cells in its on-center, while inhibiting *F*_1_ cells in its off-surround. Together, these signals embody the ART Matching Rule for object attention. The ART Matching Rule circuit ensures that category learning does not suffer from catastrophic forgetting. **(C)** If *V* mismatches *I* at *F*_1_, then the ART Matching Rule chooses a new STM activity pattern X* (the yellow pattern) at cells where the bottom-up and top-down patterns match. Mismatched features (white area) are inhibited. If the pattern X* of attended features across *F*_1_ represents a big enough mismatch to activate the orienting system (that is, if *ρ/I/* – /*X/* > 0), then a novelty-sensitive nonspecific arousal burst is activated there (cf. the N200 in Figure 10), which resets, or inhibits, the currently active category *Y* and drives hypothesis testing and memory search until another category is chosen **(D)** that supports a good enough match to keep the orienting system quiet while category learning occurs. If, however, in **(B)** a good enough match occurs between *I* and *V* to keep the orienting system quiet, then it reactivates the pattern *Y* at *F*_2_ which, in turn, reactivates X* at *F*_1_. Positive feedback loop hereby dynamically links, or binds, X* with *Y*. In both of these latter cases, a feature-category resonance focuses attention on the active critical feature pattern while learning it in both the bottom-up adaptive filter and top-down learned expectation (adapted with permission from Carpenter and Grossberg, 1987a).

Vigilance control is an important process in regulating the concreteness or abstractness of the categories that can be learned by an ART model (Figure 7), whether these categories are cognitive, spatial, or motor. ART regulates how spatial patterns of activity, or short-term memory (STM) traces, across a network of cells respond on a fast time scale to external and internal inputs, as they interact with adaptive weights, or long-term memory (LTM) traces, to learn recognition categories without experiencing catastrophic forgetting.

The learning of recognition categories in ART builds upon the laws of a Self-Organizing Map, or SOM (Grossberg, 1976a, 1978, 1980; Kohonen, 1984), in which LTM traces multiply, or gate, bottom-up signals from a feature level *F*_1_ to a category level *F*_2_ before the gated signals compete to choose and store in STM the cell activity, or small number of cell activities, that received the largest inputs (Figure 7A). These active *F*_2_ cells drive learning in the LTM traces that abut them, thereby tuning the LTM traces and causing the winning *F*_2_ cells to become recognition categories that respond selectively to sets of similar feature patterns across *F*_1_.

ART models how top-down attentive matching (Figure 7B) dynamically stabilizes the learning by LTM traces both within bottom-up adaptive filters from level *F*_1_ to *F*_2_ *and* within top-down learned expectations from level *F*_2_ to *F*_1_, as described more fully below and in the caption of Figure 7.

The modulatory on-center of a top-down expectation learns a prototype that focuses attention upon the spatial pattern of *critical features* that controls predictive success. When such an expectation is read out in the absence of a bottom-up input pattern, the modulatory on-center of the ART Matching Rule can prime, or sensitize, feature-selective cells in the on-center, without firing them to suprathreshold activity levels. Bottom-up input patterns that match the on-center well enough are needed to fire on-center cells to suprathreshold activation levels, and to thereby begin the process of focusing attention upon the matched subset of critical features that are in the learned prototype of the expectation. While this matching process takes hold, the off-surround can fully suppress the cells that it inhibits, thereby preventing irrelevant cues from being learned and, along with it, catastrophic forgetting. Carpenter and Grossberg (1987a) proved mathematically that the ART Matching Rule is necessary to dynamically stabilize the memories of learned categories.

The bottom-up adaptive filters and recurrent competition that control learning of grid cells and place cells in Figure 2 are also SOMs, albeit SOMs that drive spatial category learning. As I noted above, within the GridPlaceMap model, the laws and parameters of each SOM in its network hierarchy are the same. The fact that grid cells and place cells learn such different receptive field properties is due to their different inputs: Arrays of stripe cells drive the learning of grid cells, while place cell learning is driven by inputs from arrays of emerging grid cells, even before their receptive fields are fully formed, leading to the beautiful receptive fields in Figures 1B,C. As in cognitive ART models, the top-down expectations in Figure 6 also obey the ART Matching Rule of Figure 7B in order to dynamically stabilize learned grid cell and place cell receptive field properties.

### 3.4. Cholinergically Modulated Vigilance Control in Cognitive Category Learning and Maintenance

**FIGURE 8:**
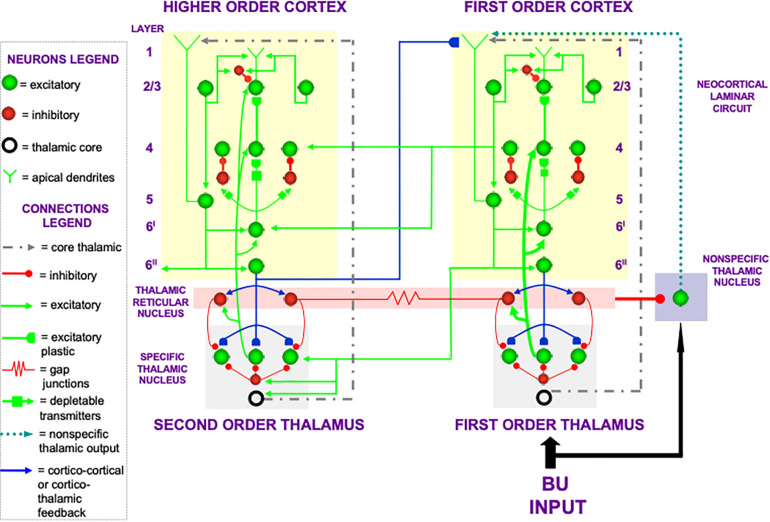
The Synchronous Matching ART, or SMART, model describes how spiking neurons in a laminar cortical hierarchy interact with specific and nonspecific thalamic nuclei to learn perceptual or cognitive categories (reprinted with permission from Grossberg and Versace, 2008).

**FIGURE 9:**
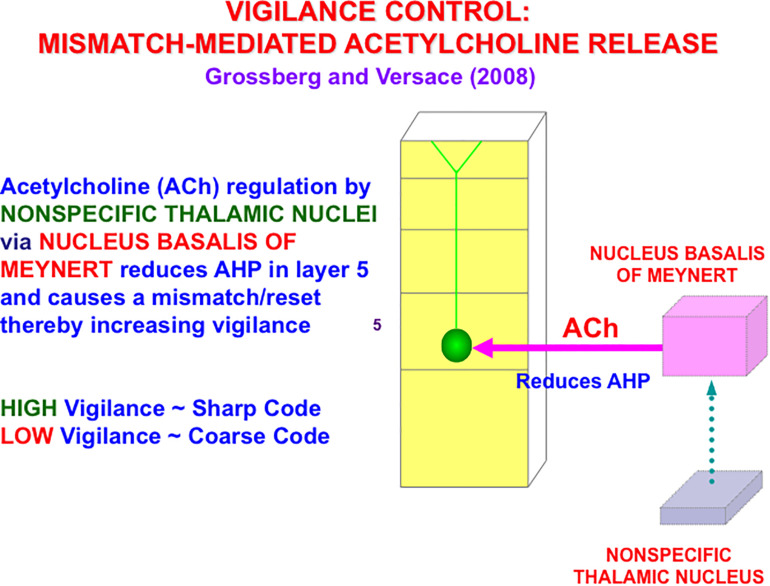
A big enough thalamocortical and corticocortical mismatch activates the nonspecific thalamic nucleus, which in turn activates the nucleus basalis of Meynert. The nucleus basalis then and releases acetylcholine (ACh) into deeper layers, notably layer 5, of multiple cortical areas. ACh release increases vigilance by reducing afterhyperpolarization (AHP) currents (reprinted with permission from Grossberg and Versace, 2008).

In the cognitive domain, vigilance control is realized by identified neurons in laminar cortical circuits that have been modeled by the Synchronous Matching ART, or SMART, model refinement of ART (Grossberg and Versace, 2008; Palma et al., 2012a, b; Grossberg et al., 2016). The SMART model is realized by spiking neurons that obey neurophysiologically measured parameters which interact within the detailed laminar cortical and subcortical circuits in Figure 8 in response to bottom-up (BU) inputs. SMART explains how vigilance may be altered by acetylcholine release when the nucleus basalis of Meynert is activated via the nonspecific thalamus (Figure 9; van der Werf et al., 2002). The nonspecific thalamus is itself activated by corticothalamic mismatches that occur within a specific thalamic nucleus during an ART hypothesis testing and learning cycle (Figures 7, 8).

Figure 9 also summarizes how activation of the nucleus basalis of Meynert broadcasts cholinergic signals to layer 5 cells. The released acetylcholine inhibits afterhyperpolarization (AHP) currents, thereby increasing cell excitability and increasing vigilance.

The SMART model further develops a series of ART category learning and recognition models that have been developed over the years to include and explain increasingly detailed and system-wide thalamocortical and corticocortical interactions (e.g., Grossberg, 1976a, b, 2003, 2013a, 2017a, b, 2020, 2021; Carpenter and Grossberg, 1987a, b; Carpenter et al., 1989, 1991a,b, 1992; Grossberg et al., 1997a; Ames and Grossberg, 2008; Grossberg and Versace, 2008; Grossberg and Huang, 2009; Cao et al., 2011; Chang et al., 2014).

**FIGURE 10:**
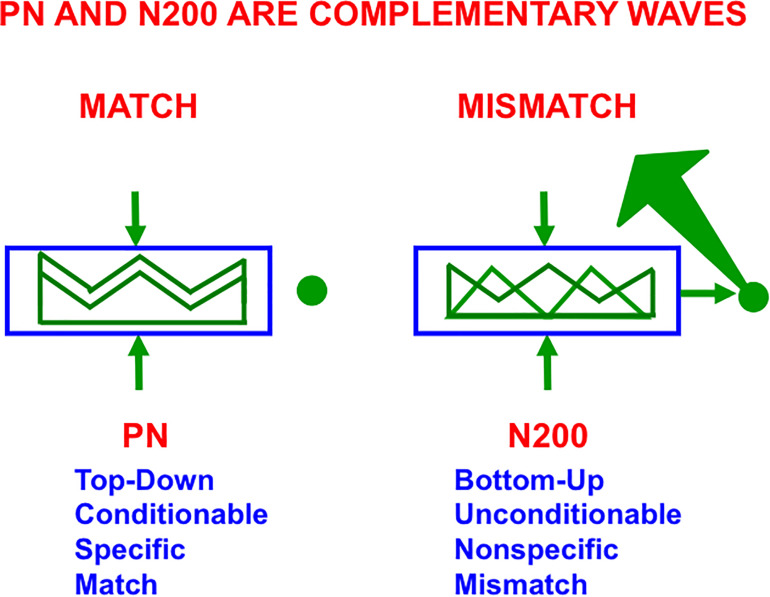
The Processing Negativity, or PN, event-related potential obeys computationally complementary laws to the N200 event-related potential (see the text for details; reprinted with permission from Grossberg, 2017b).

As illustrated in Figure 10 (left panel), in all ART models, including SMART, a good enough top-down match with a bottom-up feature pattern triggers a *feature-category resonance* that drives learning of a new recognition category, or refinement of an already established one, as well as conscious recognition of the object that the category codes (Grossberg, 2017b). Both ART and SMART explain and simulate how sensory and cognitive information processing may be broken into cycles of match and mismatch (Figures 7, 10), or resonance and reset, which will be seen below to correspond to cycles of faster gamma oscillations (40–60 Hz) and slower beta oscillations (16–20 Hz); see Buffalo et al. (2011) and Terporten et al. (2019). When theta rhythms (4–12 Hz) organize these cycles through time, then theta-modulated gamma oscillations and beta oscillations are the natural result.

### 3.5. Breakdowns in Vigilance Control of ART Dynamics Clarifies Mental Disorders

Within SMART, increased layer 5 excitability to predictive mismatches due to acetylcholine release may cause reset of a currently active category via the layer 5-to-6^I^-to-4 circuit that realizes part of the model’s ART Matching Rule (Figure 8). Such a reset can occur even if top-down feedback sufficiently matched a bottom-up input to avoid reset just moments before. An acetylcholine increment hereby drives a search for finer recognition categories, even when bottom-up and top-down signals have a pretty good match based on similarity alone.

How perceptual and cognitive vigilance control is modulated by the nucleus basalis of Meynert and acetylcholine has wide-ranging implications. For example, breakdowns of vigilance control that undermine the ART Matching Rule circuit via layer 5 can, depending on whether phasic or tonic vigilance control, or both, are affected, cause symptoms of Alzheimer’s disease, autism, amnesia, and disrupted slow wave sleep (Grossberg, 2017a).

### 3.6. Gamma and Beta Oscillations Covary With PN and N200 Event-Related Potentials

ART and its variants such as SMART are supported by neurophysiological data on multiple organizational levels. For example, when a match is good enough to generate a feature-category resonance in SMART, it is supported by fast gamma oscillations, whereas a big enough mismatch triggers slower beta oscillations (Grossberg and Versace, 2008; Grossberg, 2009). Grossberg (2013a) reviews neurophysiological experiments that support this prediction, whether during different effects of attention on different layers in the primary visual cortex (Buffalo et al., 2011), spatial attention shifts in frontal eye fields (Buschman and Miller, 2007), or learning of new place cells in the hippocampus (Berke et al., 2008). Synchrony is a characteristic feature of the cells that participate in gamma frequency oscillations (Freeman, 1975; Eckhorn et al., 1988; Gray and Singer, 1989; Pollen, 1999; Engel et al., 2001; Buschman and Miller, 2007; Gregoriou et al., 2009), whether during preattentive perceptual grouping or attentive category learning and recognition (Grossberg, 1980; Grossberg and Somers, 1991; Maldonado et al., 2000; Yazdanbakhsh and Grossberg, 2004).

On a coarser level of neurophysiological measurement, a ProcessingNegativity, or PN, event-related potential or ERP (Näätänen et al., 1978) can be recorded by scalp electrodes during the match of a bottom-up feature pattern with a top-down learned expectation that triggers a gamma oscillation (Hermann and Knight, 2001). In contrast, a big enough mismatch can activate the ART orienting system to drive hypothesis testing and memory search for a new, or better matching, category (Figure 7C), leading to a good enough match to again drive category learning. Such an activation of the orienting system is accompanied by a beta oscillation (Haenschel et al., 2000) and an N200 ERP (Näätänen et al., 1982). As summarized in Figure 10, the PN and N200 ERPs exhibit computationally complementary properties (Grossberg, 2000, 2017b).

### 3.7. What Happens to Place Cells When Grid Cells Collapse?

Many additional facts and explanations of how the extrinsic theta rhythm and vigilance control work in cognitive, motor, and spatial processing regions are found in Grossberg (2021). As just one example, if a septal lesion eliminates the extrinsic theta rhythm and causes grid cells to become disorganized, some place cells may remain intact. Since place cells are the hippocampal spatial categories that can represent spaces large enough to control spatial navigation, then why are grid cells needed at all? When grid cells collapse, place cells cannot represent the large spaces that are the least common multiple of the grid cell scales that previously input to them. These are, however, the place cells that enable us to navigate in many of the spaces that are ecologically important for our survival.

In addition, if grid cells collapse, then the feedback loop in Figure 6 that dynamically stabilizes both grid cell and place cell receptive fields is broken, leading to unstable representations of space at even the level of place cells. Indeed, Kentros et al. (2004) noted that “conditions that maximize place field stability greatly increase orientation to novel cues. This suggests that storage and retrieval of place cells is modulated by a top-down cognitive process resembling attention and that place cells are neural correlates of spatial memory.” The role of top-down attentive feedback in stabilizing place cell receptive fields is also supported by results of Morris and Frey (1997), who reported that hippocampal plasticity reflects an “automatic recording of attended experience.” The importance of learning in these changes is shown by the fact that NMDA receptors mediate long-lasting hippocampal place field memory in novel environments (Kentros et al., 1998).

Some place cells develop before grid cells do. They could be learned directly from stripe cells, say via the direct pathway from entorhinal cortex to CA1 (Figure 6). This fact may clarify how rat pups behave before they are around two-and-one-half weeks old. Until then, rat pups tend to stay in or near their nests. Exploration of larger spaces occurs around the same time when grid cells rapidly develop (Langston et al., 2010; Wills et al., 2010, 2012; Muessig et al., 2015), and drive learning of place cells capable of controlling navigation in these larger spaces.

### 3.8. Disinhibition of the Septal Theta Rhythm via the Basal Ganglia

The basal ganglia play a key role in gating on or off perceptual, cognitive, emotional, and motor processes, thereby helping to coordinate them to achieve desired behavioral goals. Significant progress has been made in developing neural models of how the basal ganglia work and realize these properties (e.g., Brown et al., 1999, 2004; Grossberg, 2016; Grossberg and Kishnan, 2018). Typically, turning on the basal ganglia substantia nigra pars reticulata, or SNr, inhibits a tonically active inhibitory pathway, thereby disinhibiting the cellular targets of that inhibition and enabling the disinhibited neural pathways to fire. A classical example is how disinhibition of the deeper layers of the superior colliculus by the basal ganglia, or direct electrical simulation, enables a saccadic eye movement of prescribed direction and length to be released to a desired target location (Schiller and Stryker, 1972; Hikosaka and Wurtz, 1985; McIlwain, 1986; Munoz and Wurtz, 1995a, b; Munoz et al., 2000; Watanabe and Munoz, 2010), a process that has also been extensively modeled (Grossberg et al., 1997b; Gancarz and Grossberg, 1998; Brown et al., 2004).

In the case of the septo-hippocampal theta rhythm, a volitional signal from the basal ganglia disinhibits an endogenous oscillator that drives this theta rhythm from cells outside the hippocampus (Silkis, 2008). Available data do not seem sufficient to derive an anatomically detailed model of the microcircuit of this oscillator. Instead, to clarify how this may happen in principle, I will summarize a type of minimal oscillator that is built from classical neurobiological components, such as cells whose potentials obey the membrane equations of neurophysiology—also called shunting interactions—as they interact within a recurrent on-center off-surround anatomy.

This kind of network has been used throughout the brain to carry out multiple functions, including functions where oscillatory dynamics may not occur some of the time, but do oscillate at other times. In particular, networks that do not oscillate with some parameter settings do oscillate when these parameters are changed, notably the rate with which inhibitory interneurons respond. For example, when recurrent on-center off-surround networks include inhibitory off-surround interneurons that react more slowly than their on-center cells, then they tend to oscillate when they also receive a sufficiently large volitional signal, also called a GO signal. This kind of result was first mathematically proved and simulated in Ellias and Grossberg (1975). It has been modeled in scores of articles since by many authors.

**FIGURE 11:**
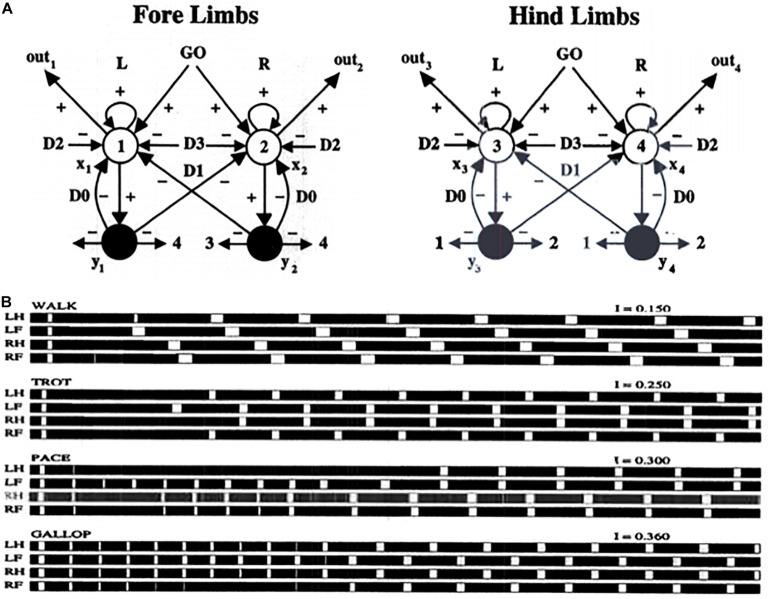
**(A)** Multiple movement gaits are generated by suitably connected recurrent shunting on-center off-surround networks when they are activated by a volitional GO signal of variable size. Abbreviations in **(B)**: LH, left hind; LF, left front; RH, right hind; RF, right front (see the text for details; reprinted with permission from Pribe et al., 1997).

Specializations of this oscillator have been used to model a number of basal-ganglia disinhibited oscillatory dynamics, including the control of mammalian movement gaits (Pribe et al., 1997). Figure 11A describes a model central pattern generator, or CPG, that is capable of generating multiple gaits using a pair of recurrent on-center off-surround networks, one to control the forelimbs and the other to control the hindlimbs of a quadruped animal. The cells in the CPG obey shunting interactions. The gaits are activated by a volitional GO signal that sends signals to both the forelimb and the hindlimb circuits. This excitatory GO signal represents a simple way to model basal ganglia disinhibition. The need for a signal with this functionality was clear in modeling studies decades before detailed models of the basal ganglia were developed.

In the CPG of Figure 11A, increasing the GO signal increases the frequency of the gait, and also leads to bifurcations into different gaits that are stable at higher speeds. Excitatory neurons are denoted by open disks. Inhibitory interneurons are denoted by black disks. Self-inhibitory inhibition is labeled by parameter D0, inhibition between forelimbs and between hindlimbs is labeled by D1, inhibition between matched forelimbs and hindlimbs is labeled by D2, and inhibition between crossed forelimbs and hindlimbs is labeled by D3. Figure 11B summarizes computer simulations of four gaits (walk, trot, pace, and gallop) that arise due to different GO signal levels as an emergent property of network interactions. Variations of the model also generate the primary human gaits (walk, run) and elephant gaits (amble, walk).

The above type of circuit clarifies how increasing basal ganglia disinhibition of the septum can increase the theta rhythm frequency. It also clarifies how basal ganglia disinhibition of gait-controlling circuits in other parts of the brain (Takakusaki, 2017) can increase running speed. Many experiments have been done over the years to clarify how theta rhythms, in concert with closely related respiratory rhythms (Cao et al., 2012), can regulate the energizing, coordination, and learning of multiple actions, beyond the classically reported correlations between theta-modulated sniffing and whisking (Komisaruk, 1970; Macrides and Chorover, 1972; Semba and Komisaruk, 1984; Ranade et al., 2013) to a wide range of other brain regions and learned behaviors (Landfield et al., 1972; Macrides et al., 1982; DeCoteau et al., 2007; Kaplan et al., 2012; Kleinfeld et al., 2014, 2016; Grion et al., 2016; Rojas-Líbano et al., 2018).

### 3.9. The Olfactory Code as a Temporal Series of Spatial Patterns at a Theta Oscillatory Rate

Early experiments on theta-modulated sniffing were carried out by Walter Freeman. In these early classical experiments, Freeman (1975) discovered a resonance phenomenon by performing parallel electrode experiments on the cat prepyriform cortex. When a cat, or rabbit, smells an expected scent, its cortical potentials are amplified until a synchronized oscillation of activity is elicited across the cortical tissue. The oscillation organizes the cortical activity into a *temporal sequence of spatial patterns*. The spatial patterns of activity across cortical cells carry the olfactory code. The temporal series of spatial patterns drives the spatial patterns from the sensory periphery into higher sensory and cortical areas while continuing to bind the signals together in a spatial pattern code.

The concept that a distributed spatial pattern of activation across a network of cells constitutes a sensory neural code, whether or not it was energized by oscillatory dynamics, was supported even then by experiments from several labs (Pfaffman, 1955; Erickson, 1963; Freeman, 1972; Somjen, 1972). By contrast, when the cat or rabbit smells an unexpected scent, then cortical activity is markedly suppressed. Freeman traced differences in cortical activity after expected vs. unexpected scents to gain changes within the cortical tissue. Grossberg (1973, 1976a, 1976b) showed how such gain changes, due to shunting dynamics, could also explain the tendency for the most active populations to phase-lead less active populations.

Freeman (1975) also reported the experiments of Dumenko (1968, 1970) on dogs who were trained to perform a conditioned response to both visual and auditory stimuli, leading to a high correlation between the EEGs of their visual and auditory cortices in the theta range 5–7 Hz.

These neurophysiological studies supported theorems about biological neural networks showing that the functional unit of fast neural information processing is a distributed *spatial pattern* of activity, or STM traces, across a network, and that the functional unit of learning is a distributed *spatial pattern* of adaptive weights, or LTM traces, across the network (Grossberg, 1968a, b, 1969, 1970, 1971, 1980), whether the energetic carrier of these patterns converges to an equilibrium point or rhythmically oscillates through time.

### 3.10. Toward Comprehensive Neural Architectures

It remains to develop a comprehensive neural model of how the entorhinal-hippocampal circuits that are described above work together with other brain regions to release, learn, gain-control, and coordinate sequences of goal-oriented naturalistic behaviors. Some neural architectures, such as the SOVEREIGN family of architectures (Gnadt and Grossberg, 2008; Grossberg, 2019), model how a brain can incrementally learn planned action sequences to navigate toward a rewarded goal, and can do so while being modulated by basal ganglia gating. The SOVEREIGN model’s ability to do so was tested in a 3D virtual reality maze that the animat learned to navigate in order to acquire a reward.

The SOVEREIGN2 architecture embodies interactions between more of the brain processes whose interactions are needed to realize biological intelligence. These processes have previously been modeled in multiple articles over the past 40 years. The Abstract of Grossberg (2019) summarizes these processes:

“Key new perceptual, cognitive, cognitive-emotional, and navigational processes require feedback networks which regulate resonant brain states that support conscious experiences of seeing, feeling, and knowing. Also included are computationally complementary processes of the mammalian neocortical What and Where processing streams, and homologous mechanisms for spatial navigation and arm movement control. These include: Unpredictably moving targets are tracked using coordinated smooth pursuit and saccadic movements. Estimates of target and present position are computed in the Where stream, and can activate approach movements. Motion cues can elicit orienting movements to bring new targets into view. Cumulative movement estimates are derived from visual and vestibular cues. Arbitrary navigational routes are incrementally learned as a labeled graph of angles turned and distances traveled between turns. Noisy and incomplete visual sensor data are transformed into representations of visual form and motion. Invariant recognition categories are learned in the What stream. Sequences of invariant object categories are stored in a cognitive working memory, whereas sequences of movement positions and directions are stored in a spatial working memory. Stored sequences trigger learning of cognitive and spatial/motor sequence categories or plans, also called *list chunks*, which control planned decisions and movements toward valued goal objects. Predictively successful list chunk combinations are selectively enhanced or suppressed via reinforcement learning and incentive motivational learning. Expected vs. unexpected event disconfirmations regulate these enhancement and suppressive processes. Adaptively timed learning enables attention and action to match task constraints. Social cognitive joint attention enables imitation learning of skills by learners who observe teachers from different spatial vantage points.”

However, neither SOVEREIGN nor SOVEREIGN2 describes how all of these processes are coordinated by theta and respiratory rhythms. This should be a major goal of future research that builds upon this foundation.

## 4. Intrinsic Theta Rhythm Dissociates Learned Read-Out and Read-In

Part of this research would need to include the existence, neural mechanism, and functional roles of an intrinsic source of a theta rhythm which, unlike the septally mediated theta rhythm, is atropine-resistant and arises entirely within the hippocampal CA1 region (Kramis et al., 1975; Goutagny et al., 2009). Intrinsic and extrinsic theta oscillations can interact in complex ways (e.g., Montgomery et al., 2009) as part of the tri-synaptic hippocampal organization of CA1, CA3, and the dentate gyrus (Figure 6; Amaral and Witter, 1989; Amaral, 1993; Knierim, 2015).

Hippocampal area CA1 helps to support functions other than spatial navigation, including learning, recognition, and memory that involve visual, cognitive, and emotional functions (e.g., Zola-Morgan et al., 1986; Rempel-Clower et al., 1996; Leutgeb et al., 2004; Manns et al., 2007; Dong et al., 2009). As noted in Section 1.6, LEC seems to process cue-related information, including its adaptive timing in behavior, whereas MEC processes the kind of spatial information that is computed by grid cells and place cells. Both kinds of information may be processed through CA1, albeit in different cortical layers (Soltesz and Losonczy, 2018).

Such a sharing of processing resources is understandable from the perspective that “these areas, which receive multimodal inputs from various sensory and motor regions, represent the end-points of the what and where streams of visual processing, which are then integrated at the hippocampus” (Saleem, 2019, p. 73). For example, visual cues are used for visual object recognition, as well as to define landmarks with which to guide spatial navigation. Likewise, the commands for navigating in a fixed direction and distance to reach a desired goal object using *object vector cells* (Deshmukh and Knierim, 2011; Saleem, 2019), and for reaching a nearby object without navigational movement using *motor difference vectors*, both involve similar computations, albeit in different parts of the brain (e.g., Georgopoulos et al., 1981, 1982; Bullock and Grossberg, 1988; Gnadt and Grossberg, 2008). This extension of the classical What and Where cortical “two stream hypothesis” (Mishkin, 1982; Mishkin et al., 1983; Goodale et al., 1991; Goodale and Milner, 1992) has been further developed and refined by several authors (e.g., Hoang et al., 2018; Nilssen et al., 2019).

Below the discussion of CA1 will begin with an analysis of how an intrinsic theta rhythm enables large numbers of converging signals to be processed and tuned by learning on the dendrites of their target cells.

### 4.1. Preventing Saturation of Learned Synapses by Dissociating Read-Out and Read-In

**FIGURE 12:**
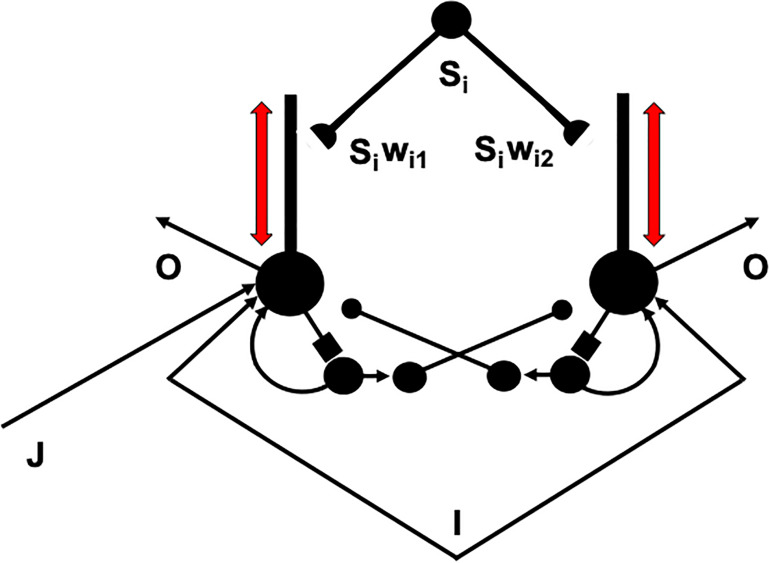
A recurrent associative dipole, or READ, circuit is a recurrent shunting opponent processing network with habituative transmitter gates. The large closed disks represent opponent ON and OFF cells. The vertical rectangular bars that abut from them represent dendritic trees. Excitatory network pathways end in arrows. Inhibitory pathways end in closed disks. Habituative pathways end in squares. A nonspecific arousal input I equally excites the ON and OFF cells. A phasic input J activates only the ON cell in this circuit, but different phasic inputs can in general activate ON and OFF cells. Sensory cues S_i_ sample the dendrites with LTM traces w_i1_ and w_i2_ and thereby become conditioned reinforcers when their abutting dendrite is activated by a back-propagating action potential (upward red arrow). Read-out from a conditioned reinforcer travels down the dendrite to the corresponding cell body (downward red arrow) (see the text for details; adapted with permission from Grossberg and Schmajuk, 1987).

I suggest that the intrinsic theta rhythm is an emergent property within the hippocampus of how our brains solve a general learning problem: If many learning trials continue throughout life, then what prevents the learned adaptive weights at synapses from saturating; that is, from hitting their maximal values and becoming unable to learn new associative contingencies in the future? A basic neural circuit called a REcurrent Associative Dipole, or READ, circuit (Figure 12) is able to regulate cognitive-emotional learning in response to rewards and punishments throughout the lifespan without saturating the adaptive weights that input to its *opponent* affective representations (Grossberg, 1972, 1975, 1982a, 1984; Grossberg and Schmajuk, 1987; Dranias et al., 2008; Gnadt and Grossberg, 2008; Grossberg et al., 2008), such as fear vs. relief.

The READ circuit does this by carrying out a process that I call *dissociation of associative read-out from read-in*, which leads in a natural way to a theta rhythm, as explained below. Many other brain systems realize this dissociation, including within the CA1 region of the hippocampus, to solve a general learning problem that I will describe after noting some important features of the READ circuit. Hasselmo (2005) has described neurobiological data that are consistent with such a dissociation and his model of it.

### 4.2. Uniform Activity Patterns Are Suppressed by a Recurrent On-Center Off-Surround Network

The problem is that read-*out* of a previously learned adaptive weight, such as *w*_i1_ or *w*_i2_ in Figure 12, to a target cell should *not* always trigger new learning—that is, read-*in*—by that adaptive weight, even if it succeeds in activating its target cell. Correlation learning *per se* is not sufficient. To understand why automatic read-in would cause a serious problem, consider the *total activity pattern* across all the cells of a network, not just the activity of a single cell. Multiple inputs can converge on each cell across such a network from multiple cue-activated stimuli, such as the signals *S*_i_ in Figure 12. Each input can be active at different times and with different magnitudes to each cell in the network.

Consider what happens when all the inputs that converge upon the network’s cells sum up to create approximately equal *total* inputs to *all* the cells; that is, a *uniform* input pattern perturbs the network. If this state of affairs persists for a while, then all activities the network’s cells will also become approximately equal. Such a uniform pattern of activities carries no information because it does not prefer any particular feature combination that is represented by a subset of these cells to any other. A uniform activity pattern thus represents functional “noise.” It should *not* drive new learning, because if it did, then previous learning could quickly be washed away by new learning of “noise.” In order to avoid this disaster, associative read-out cannot always force new associative read-in. Somehow the “noise” must be suppressed, so that only the information in non-uniform feature patterns can be learned.

### 4.3. Competition Chooses Cells That Trigger Back-Propagating Teaching Signals

A READ circuit accomplishes noise suppression by letting contrast-enhancing competition—embodied by a recurrent on-center off-surround network—occur across the network in response to each input pattern (Figure 12), before the population that wins the competitive choice generates a teaching signal that drives new learning. If all the inputs are approximately equal, then the uniform activity pattern that they cause is suppressed by the competition, no teaching signal will be emitted, and no new learning occurs. If some inputs are sufficiently bigger than others, then their activities will be contrast-enhanced, normalized, and stored by the recurrent on-center off-surround network, while the smaller activities are suppressed by the competition. A teaching signal will be emitted only by cells whose activities win the competition. Only the winning cells will therefore be able to drive learning of the incoming input pattern.

**FIGURE 13:**
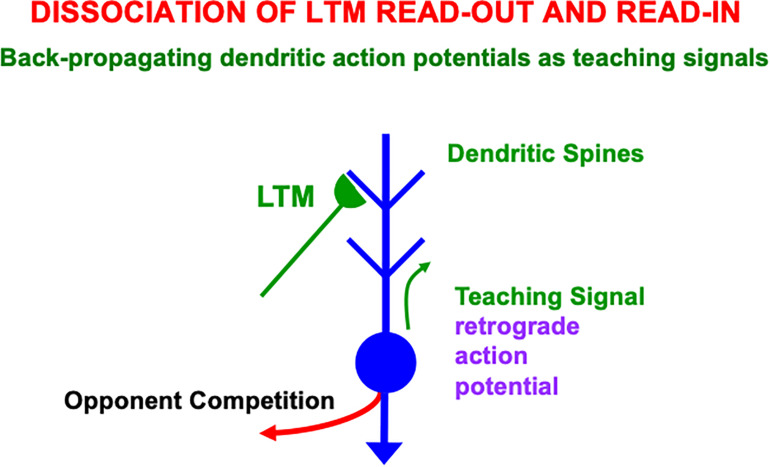
Illustration of how the ON and OFF cells in a READ circuit or, more generally, the category learning cells in a SOM, interact with cue-activated adaptive signals on their dendritic trees. Each dendritic tree possesses dendritic spines upon which a large number of cue-activated adaptive signals can converge. When a cell is active, it can generate retrograde, or back-propagating, action potentials that act as teaching signals for the adaptive weights, or long-term memory (LTM) traces, of currently active cue-activated pathways at its dendritic spines (reprinted with permission from Grossberg, 2021).

How does a winning activity trigger a teaching signal? In the READ circuit, the winning cells remain active long enough to trigger back-propagating action potentials in their dendrites (Figures 12, 13), while they maintain their activities via the recurrent on-center off-surround interactions. These back-propagating action potentials serve as teaching signals that are associated with simultaneously active input signals to the chosen dendrites (Grossberg, 1975, 1982a; Markram et al., 1995, 1997; Magee and Johnston, 1997). If, for example, signal *S*_3_ in Figure 12 is active when the dendrite of cell C_1_ is active (upward pointing red arrow), then the LTM trace *w*_31_ will learn that association, while the LTM trace *w*_32_ decreases because its abutting dendrite is then inactive.

Because the READ circuit is a recurrent on-center off-surround network whose cells obey shunting dynamics (Grossberg, 2013b), the competition computes teaching signals that are *normalized net activities* in which the smaller activity is inhibited to zero and the larger activity is sensitive to the ratio of the inputs. The LTM traces can continue to learn these normalized net values throughout life, without ever saturating.

### 4.4. A Rhythm Enables Associative Read-In and Read-Out Without a Noise-Learning Catastrophe

An electrical signal cannot go both down a dendrite and up it at the same time. Read-*out* from a previously learned LTM weight occurs when a signal goes down a dendrite to its basal cell. Read-*in* of a new LTM weight value occurs when a teaching signal goes up the dendrite from its basal cell. A single cue-activated signal *S*_i_ that persists for hundreds of milliseconds or seconds needs to be able to read-out its previously learned LTM weight *and* to read-in any new information with which it is associated. A cyclic rhythm of signaling down and up the sampled dendrites enables a sustained input signal to experience both associative read-in and read-out on its abutting dendrites, without incurring a noise-learning catastrophe. Such a dendritic rhythm is the price of avoiding noise-driven catastrophic forgetting of previously learned featural differences.

In summary, the following cyclic processes are needed to dissociate associative read-out from read-in:

1. Read-*out* of cue-activated LTM-gated signals, as from the conditionable signals *S*_i_*w*_ij_ with adaptive weights *w*_ij_, j = 1,2, in the READ circuit in Figure 12;
2. contrast-enhancing competition via the recurrent on-center off-surround feedback loop in Figure 12;
3. read-*in* of the contrast-enhanced and normalized activities into the adaptive weights *w*_ij_ in Figure 12; and
4. repeat.

### 4.5. SOM Theta Rhythm Dissociates Read-Out From Read-In With Back-Propagating Teaching Signals

Such a recurring read-out/choice/read-in cycle is assumed to also occur in self-organizing maps, or SOMs, during perceptual, cognitive, and spatial category learning (e.g., Figure 7A), not just in opponent READ circuits during reinforcement learning. A theta rhythm results in all these cases.

**FIGURE 14:**
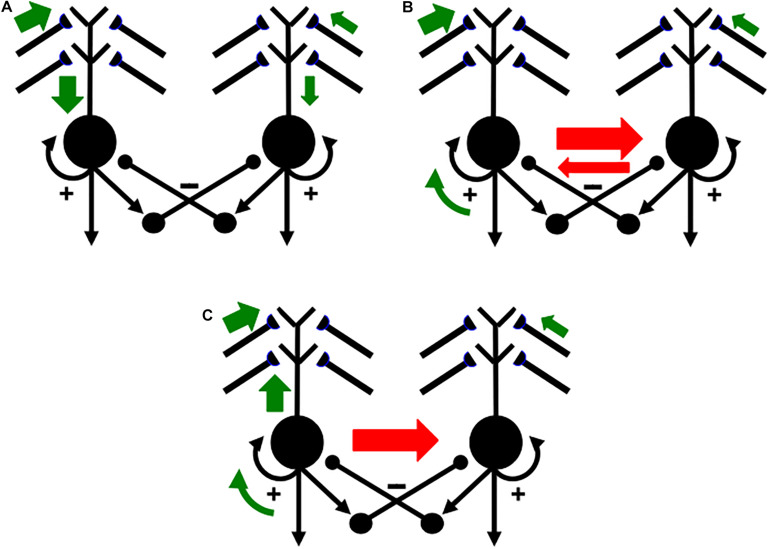
Activation and learning dynamics on the dendritic spines of cells in a READ circuit or SOM. Green arrows depict currently active excitatory signals. Red arrows depict currently active inhibitory signals. The sizes of the arrows depict the relative strength of their signals (see the text for details; reprinted with permission from Grossberg, 2021).

Figure 14 summarizes how this cycle is proposed to occur in greater detail. Figure 14A shows two competing SOM category cells that each receive multiple inputs via their dendritic trees, which are drawn above the cell bodies. Dendritic trees greatly expand the total surface area where large numbers of inputs from multiple sources can send signals that converge upon a single cell (Rall, 1959, 1962, 1995). Both entorhinal grid cells (Schmidt-Hieber et al., 2017) and hippocampal place cells (Sheffield and Dombeck, 2015) have dendritic trees. In the hippocampus, these inputs arrive from the feature detectors of the SOM spatial category learning cells, whether from stripe cells to developing grid cells, or from developing grid cells to developing place cells (Figure 2).

In Figure 14A, the left cell receives a bigger total input than the right cell (thick vs. thin green arrows). The left cell can therefore inhibit the right cell more than conversely (thick vs. thin red arrows in Figure 14B) while its activity is contrast-enhanced by its recurrent on-center (curved green arrow). The left cell can hereby win the competition with the right cell and shut it off. As this is happening, the winning left cell also generates a back-propagating action potential through its dendritic tree (upward green arrow in Figure 14C; see also Figure 12). This back-propagating action potential is a teaching signal that drives associative learning in the LTM traces of pathways that are currently inputting to that dendritic tree. These dynamics repeat themselves through time as different input patterns choose different winning category cells.

Buzsáki (2002) summarizes many facts about the theta rhythm that are consistent with the above explanation. Buzsáki notes, in particular, that “theta oscillation may provide a mechanism for bringing together in time afferent-induced depolarization of pyramidal cell dendrites and dendritic invasion of fast spikes, the key elements for the induction of synaptic plasticity” (p. 325). His discussion describes many useful biophysical facts about this kind of learning, but not in terms of the basic design problem of how it prevents the catastrophic forgetting due to massive learning of noise that would have occurred without dissociation of associative read-out from read-in.

### 4.6. Gated Dipole Fields

The cyclic nature of this process can now be better understood mechanistically. As noted above, the first reason for cyclic learning is that an electrical signal cannot go both down a dendrite (Figure 14A) and up it (Figure 14C) at the same time. This property requires a rhythmic alternation between the down-state of read-out of previously learned memories, and the up-state of read-in of new, or memory-refining, inputs. But how is this functional property achieved mechanistically?

The proposed answer, briefly stated, is by designing the competition that interpolates intervals of read-out and read-in as a *gated dipole field* (Grossberg, 1972, 1982a,b, 1984). A gated dipole field is simply a recurrent shunting on-center off-surround network whose recurrent feedback signals, notably its positive on-center feedback signals, *habituate* in an activity-dependent way, as illustrated by the habituative positive feedback signals (square synapses) in Figure 12. This activity-dependent habituation gradually weakens the signal that it gates through time. This simple refinement endows a gated dipole field with remarkable properties that are proved mathematically in the above articles, and which clarify why this network design occurs in multiple parts of the brain. Grossberg (2013b) more fully describes concepts and equations for recurrent shunting on-center off-surround networks and activity-dependent habituation.

In ART category learning circuits (Figure 7), a gated dipole field controls the recurrent competition between recognition categories at level *F*_2_ of the network. This recurrent competition chooses the category that receives the largest total input from the currently active bottom-up feature pattern (Figure 7A). When the chosen category is activated, it reads-out a top-down learned expectation. A sufficiently big mismatch between the currently active bottom-up feature pattern and top-down learned expectation pattern (Figure 7B) can activate the orienting system (Figure 7C). Such activation causes a burst of nonspecific arousal that is delivered equally to all the category learning and recognition cells at level *F*_2_, in keeping with the heuristic idea that “novel events are arousing.”

Such a nonspecific novelty burst selectively resets the currently active category at *F*_2_, leading to a memory search, or hypothesis testing, that ends by choosing a better matching category. But how does a *nonspecific* input *selectively* do *anything*? The answer is that the positive feedback signal in the on-center of the gated dipole weakens through time due to activity-dependent habituation. As a result, a nonspecific arousal burst can reset the currently active, but habituating cell, and thereby allow a better-matching category to get activated from among unhabituated category cells.

### 4.7. Gated Dipole Fields Control Perseveration, Normalization, Choice, Search, and Theta Rhythm

The same property of activity-dependent habituation that resets a currently active category in response to a nonspecific arousal burst is also responsible for preventing preservation of category activation when there is no reset. Said in another way, without activity-dependent habituation or competitive reset, the STM storage of activity by a recurrent on-center off-surround network could last indefinitely.

Activity-dependent habituation limits perseveration of the on-center activity in every gated dipole field. As one quantitatively simulated example, psychophysical data about visual persistence have been quantitatively simulated by a gated dipole network (Francis et al., 1994; Francis and Grossberg, 1996a, b). As the on-center habituates, the recurrent off-surround is disinhibited, and inhibits the previously active on-center cell. As the inhibited on-center recovers from habituation, its driving input can fire it again, after which the cycle repeats itself for as long as the input remains on.

Remarkable parsimony of design is realized by a gated dipole field because this single type of network normalizes total network activity, chooses winning category cells, limits perseveration, drives memory search, and supports a theta rhythm.

### 4.8. Why Phase Precession of the Theta Rhythm?

Using back-propagating action potentials as teaching signals also helps to explain intriguing parametric properties of the theta rhythm, such as the famous *phase precession* whereby action potentials occur on progressively earlier phases of the theta cycle as a rat traverses the place field of the recorded unit (O’Keefe and Recce, 1993; Skaggs et al., 1996). Phase precession in the GridPlaceMap model follows from two interacting properties:

First, each SOM category cell is activated through time via a receptive field whose input connections increase in strength as an animal navigates closer to the center of the receptive field. A Gaussian receptive field is a classical example.

Second, the category cells interact via shunting dynamics. As a result, larger inputs are integrated more quickly through time. Cells are thus activated earlier in each theta cycle as the center of the cell’s receptive field is approached.

In summary, key properties of phase precession occur in the GridPlaceMap model because its category cells interact via shunting recurrent on-center off-surround networks whose cells receive inputs from receptive fields whose connections increase in strength as the animal approaches each cell’s preferred position.

In addition to grid cells and place cells, several other types of cells have been reported that help to create a representation of space that animals use while they navigate. One such cell type is called a *border cell* because it fires when an animal is at or near the border of an enclosure. Solstad et al. (2008, p. 1865) reported that border cells are found throughout the MEC, as well as the parasubiculum, and “may be instrumental in planning trajectories and anchoring grid fields and place fields to a geometric reference frame.” I will defer a discussion and analysis of how border cells may form and work to another place and time.

### 4.9. Theta-Modulated Gamma Rhythms

Another interesting and functionally important emergent property of the model presented here is the existence of theta-modulated gamma rhythms, whose existence follows immediately from the occurrence in SMART (Grossberg and Versace, 2008) of gamma oscillations in a match state that are modulated by an underlying theta rhythm. Compatible data are reported by Sirota et al. (2008, p. 683) who noted: “A significant fraction of putative pyramidal cells and interneurons as well as localized gamma oscillations in all recorded neocortical areas were phase biased by the hippocampal theta rhythm. We hypothesize that temporal coordination of neocortical gamma oscillators by hippocampal theta is a mechanism by which information contained in spatially widespread neocortical assemblies can be synchronously transferred to the associative networks of the hippocampus.” These cortical areas include the primary somatosensory area and the prefrontal cortex. “These data suggest that theta oscillation entrainment provides a mechanism by which activity in spatially widespread neocortical and hippocampal networks can be temporally coordinated…during either exploration or REM sleep” (p. 693) (see also Montgomery et al., 2009; Fujisawa and Buzsáki, 2011; Ito et al., 2018).

This conclusion carries the classical proposal of Freeman (1975) about the role of theta in organizing sensory information processing into temporal series of spatial patterns into the more modern context of theta-modulated gamma and beta oscillations occurring during periods of match and mismatch, or resonance and reset. Colgin (2015) has additionally described how such a theta-gamma coupling arises during a wide range of other behavioral paradigms, including word recognition, delayed non-match-to-place, and visual processing by cortical areas V1 and V2.

## 5. Adaptively Timed Regulation of Category Learning: Theta, Gamma, and Beta Oscillations

The current article reviews, unifies, and extends neural modeling concepts that clarify how and why both extrinsic and intrinsic theta rhythms occur. Earlier modeling articles proposed how the hippocampus interacts with multiple brain regions to carry out various cognitive, emotional, and adaptive timing processes, and how these processes interact together during learning experiences. Some of these models have been discussed above, including the Adaptive Resonance Theory, or ART, models for cognitive, motor, and spatial category learning and the SOVEREIGN models for incrementally learning planned action sequences to navigate toward rewarded goals. SOVEREIGN2 summarizes a much larger number of brain processes that have been modeled over the years. These processes have yet to be synthesized within a mathematically rigorous comprehensive neural architecture.

### 5.1. Two Distinct, but Interacting, Hippocampal Mechanisms of Memory Consolidation

**FIGURE 15:**
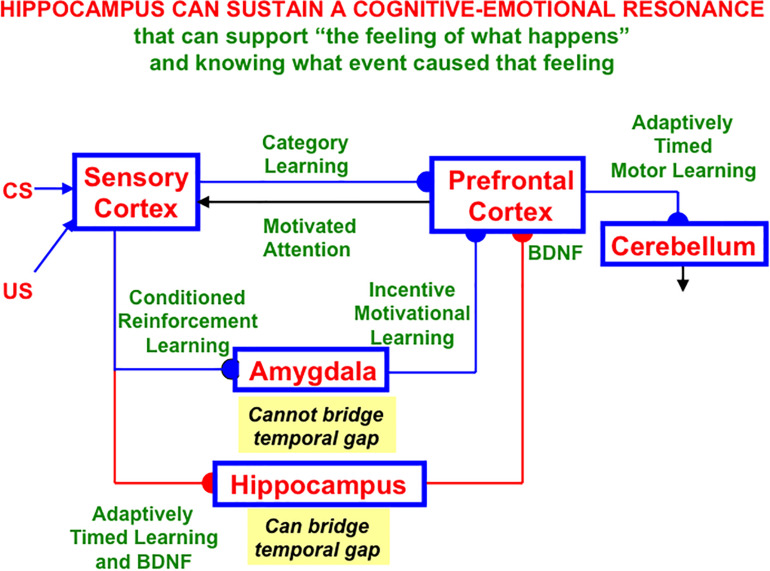
The neurotrophic START, or nSTART, model. Multiple types of learning and neurotrophic mechanisms of memory consolidation cooperate in these circuits to generate adaptively timed responses. Connections from sensory cortex to orbitofrontal cortex support category learning. Reciprocal connections from orbitofrontal cortex to sensory cortex support attention. Habituative transmitter gates modulate excitatory conductances at all processing stages. Connections from sensory cortex to amygdala connections support conditioned reinforcer learning. Connections from amygdala to orbitofrontal cortex support incentive motivation learning. Hippocampal adaptive timing and brain-derived neurotrophic factor (BDNF) bridge temporal delays between CS offset and US onset during trace conditioning acquisition. BDNF also supports long-term memory (LTM) consolidation within sensory cortex to hippocampal pathways and from hippocampal to orbitofrontal pathways. The pontine nuclei serve as a final common pathway for reading-out conditioned responses. Cerebellar dynamics are not simulated in nSTART. arrowhead = excitatory synapse; hemidisk = adaptive weight; square = habituative transmitter gate (reprinted with permission from Grossberg, 2021).

The START, or Spectrally Timed ART, model and its neurotrophically modulated refinement, nSTART (Figure 15; Grossberg and Merrill, 1992, 1996; Franklin and Grossberg, 2017), deserve special mention because they clarify how the hippocampus can coordinate ART category learning and memory consolidation with the adaptively timed learning, performance, and memory consolidation that are regulated by the LEC and the hippocampus to which it projects, all the while modulated by a theta rhythm.

The first mechanism of memory consolidation uses the ART hypothesis testing and category learning cycle (Figure 7) in the form of a cycle of resonance and reset. As explained above, the orienting system is activated by a sufficiently bad mismatch between the bottom-up sensory input pattern that activates the feature level *F*_1_ and the top-down learned expectation that is read-out from category level *F*_2_. This mismatch reduces inhibition of the orienting system, as in Figure 7B, which begins a memory search for a better matching category by triggering a nonspecific arousal burst to *F*_2_, as in Figure 7C. The hippocampus is part of this novelty-sensitive orienting system. As objects and events become familiar due to the learning cycle, they can directly access and resonate with their best matching category without any memory search. The familiar object or event has hereby learned a self-stabilizing memory, which illustrates a form of dynamically maintained memory consolidation.

As simulated by the SMART model (Grossberg and Versace, 2008), the cycle of resonance and reset is embodied by a cycle of gamma oscillations and beta oscillations. As noted in Section 3.4, although theta oscillations have not yet been quantitatively simulated during this gamma-beta cycle, the manner in which a theta rhythm organizes sequences of brain states leads naturally to the existence of theta-modulated gamma rhythms that are interrupted by intervals of beta rhythm during hypothesis testing and memory search.

Carpenter and Grossberg (1993) and Grossberg (2013a) have proposed how these memory consolidation properties can qualitatively explain data about medial temporal amnesia when the model hippocampus is ablated, thereby eliminating memory search before consolidation can occur. Such an ablation leads to properties of unlimited anterograde amnesia, limited retrograde amnesia, perseveration, difficulties in orienting to novel cues, a failure of recombinant context-sensitive processing, and different degrees of learning by amnesic and normal individuals on easy vs. demanding categorization tasks. Lesions in CA1 during transient global amnesia are correlated with a corresponding deterioration of the theta rhythm (Park et al., 2016).

In nSTART, neuromodulation by Brain Derived Neurotrophic Factor, or BDNF, sustains cortico-cortical resonances that strengthen partial learning based on previous sensory inputs. Here both the hippocampus and the cerebellum participate in adaptively timed learning (Grossberg and Merrill, 1996). The hippocampus maintains adaptively timed incentive motivational signals between active cells in the sensory cortex and the prefrontal cortex, notably orbitofrontal cortex, when visual categories are being learned. The adaptively timed hippocampal support enables thalamocortical and corticocortial category learning (see horizontal pathway from sensory cortex to prefrontal cortex in Figure 15) to occur between events that are separated in time.

Such temporal separation occurs during trace conditioning when a conditioned stimulus (CS), such as a light or tone, shuts off hundreds of milliseconds before a rewarding or punishing unconditioned stimulus (US), such as a shock or food, turns on. This requires that a CS-activated memory trace be sustained during the inter-stimulus interval in order to learn an adaptively timed association between CS and US that enables the CS to elicit a CR. As these categories are learned, the hippocampus maintains inhibition of the orienting system (not shown), so that motivated attention can be maintained throughout the adaptively timed category learning interval. The prefrontal cortex can also, at the same time, learn to activate adaptively timed actions via the cerebellum. When category learning and performance is complete, the hippocampus is disengaged, memory consolidation has occurred, and the CS alone can elicit an adaptively timed conditioned response (CR). Franklin and Grossberg (2017) review data properties that nSTART simulates when consolidation is prevented. Different properties of early vs. late hippocampal lesions are challenging to explain because no training occurs after conditioning and before hippocampal ablation.

Data about theta modulation have been reported in autistic individuals (Lushchekina et al., 2014). The imbalanced START, or iSTART model (Grossberg, 2017a) shows how, if START becomes imbalanced in particular ways, then autistic symptoms arise (Grossberg and Seidman, 2006). iSTART proposes that various individuals with autism have their vigilance stuck at an abnormally high value due to abnormal cholinergic activity, leading to abnormal orienting and memory search, learning of hyperspecific recognition categories, and a narrow focus of attention. Abnormal iSTART orienting causes abnormal beta oscillations, which have been reported (Buard et al., 2018; de Vega et al., 2019). The breakdown of a fluent response to changing environmental challenges by autistic individuals would be expected to alter their theta rhythms. Indeed, lower levels of theta spectral power and mean coherence then occur (Lushchekina et al., 2014).

## 6. Concluding Remarks

This article summarizes neural models of cortico-hippocampo-cerebellar dynamics and of the gamma, beta, and theta rhythms that underlie these dynamics during both normal and abnormal conditions.

## Author Contributions

The author confirms being the sole contributor of this work and has approved it for publication.

## Conflict of Interest

The author declares that the research was conducted in the absence of any commercial or financial relationships that could be construed as a potential conflict of interest.
